# A systematic review of randomized controlled trials of mHealth interventions against non-communicable diseases in developing countries

**DOI:** 10.1186/s12889-016-3226-3

**Published:** 2016-07-15

**Authors:** Victor Stephani, Daniel Opoku, Wilm Quentin

**Affiliations:** Department of Healthcare Management, Technische Universität Berlin, Berlin, Germany

## Abstract

**Background:**

The reasons of deaths in developing countries are shifting from communicable diseases towards non-communicable diseases (NCDs). At the same time the number of health care interventions using mobile phones (mHealth interventions) is growing rapidly. We review studies assessing the health-related impacts of mHealth on NCDs in low- and middle-income countries (LAMICs).

**Methods:**

A systematic literature search of three major databases was performed in order to identify randomized controlled trials (RCTs) of mHealth interventions. Identified studies were reviewed concerning key characteristics of the trial and the intervention; and the relationship between intervention characteristics and outcomes was qualitatively assessed.

**Results:**

The search algorithms retrieved 994 titles. 8 RCTs were included in the review, including a total of 4375 participants. Trials took place mostly in urban areas, tested different interventions (ranging from health promotion over appointment reminders and medication adjustments to clinical decision support systems), and included patients with different diseases (diabetes, asthma, hypertension). Except for one study all showed rather positive effects of mHealth interventions on reported outcome measures.

Furthermore, our results suggest that particular types of mHealth interventions that were found to have positive effects on patients with communicable diseases and for improving maternal care are likely to be effective also for NCDs.

**Conclusions:**

Despite rather positive results of included RCTs, a firm conclusion about the effectiveness of mHealth interventions against NCDs is not yet possible because of the limited number of studies, the heterogeneity of evaluated mHealth interventions and the wide variety of reported outcome measures. More research is needed to better understand the specific effects of different types of mHealth interventions on different types of patients with NCDs in LaMICs.

**Electronic supplementary material:**

The online version of this article (doi:10.1186/s12889-016-3226-3) contains supplementary material, which is available to authorized users.

## Background

As a result of increasing life-expectancy and growing welfare in low and middle income countries (LaMICs), there is a steady shift away from communicable to non-communicable diseases (NCDs) [1–3]. NCDs pose a major threat to public health in LaMICs. In 2010, NCDs already accounted for half of Disability Adjusted Life Years (DALYs) lost and for 58 % of all deaths in these countries [4]. It is predicted that this number will increase to 70 % of all deaths in 2020 [5]. The economic cost of the NCDs burden for LaMICs are estimated to reach US$21 trillion by 2030 [3].

The ability of LaMICs to provide treatment and care for the increasing number of patients with NCDs is limited by insufficient health care infrastructure, especially in rural areas [6]. At the same time there is a rapidly growing, hidden infrastructure: 90 % of the world’s population now lives within reach of a mobile phone signal [7] and the developing world has the fastest-growing cellphone subscriber market in the world [8, 9] with a mobile-cellular subscription rate of almost 90 % in 2013 [10].

The number of health care interventions using mobile phones (short mHealth interventions) is growing rapidly [11]. In particular in LaMICs, mHealth is perceived to have great potential for improving health care provision for both communicable and non-communicable diseases [12]. Most of the available literature on mHealth interventions is focused on communicable diseases (such as HIV and Malaria) or on maternal care [13]. However, the number of studies focusing on mHealth for patients with NCDs has considerably increased over the last few years. In fact, two thirds of all articles on the topic have been published between 2012 and 2015 (based on a Web of Science search with the keywords TS = (mHealth OR “mobile Health” or tele*) AND TS = (“developing”) AND TS = (NCD OR “non-communicable diseases”). Yet, evaluations of mheatlh interventions often do not follow rigorous scientific standards of randomized controlled trials (RCTs), and consequently, they carry a relatively high risk of bias [14].

Two reviews are available that have included studies analyzing certain aspects of mHealth interventions for NCDs in LaMICs: Beratarrechea et al. [15] evaluated text and automated voice interventions for chronic diseases in the developing world and Bloomfield et al. [16] performed a review of mHealth interventions against NCDs focusing only on Sub-Saharan African countries. However, as Beratarrechea et al. [15] did not focus specifically on NCDs and because Bloomfield et al. [16] focused exclusively on Sub-Saharan Africa, a comprehensive overview of the effectiveness of mHealth interventions for improved treatment and care of patients with NCDs living in LaMICs remains unavailable.

The aim of this study was 1) to systematically review the available evidence generated by randomized controlled trials (RCTs) of mHealth interventions for people with NCDs living in LaMICs, and 2) to assess the relationship between intervention characteristics and reported health-related outcomes. We focused on RCTs since they remain the gold standard for evidence of effectiveness of health interventions [17].

## Method

### Inclusion criteria

Studies were included for this review if they met the following inclusion criteria:

- The study reported results of an RCT, as defined by JN Matthews [18]
- The trial took place in at least one country that was classified as an LaMIC as defined by the World Bank classification of country income groups [19]
- The intervention involved the use of mHealth as defined by the Global Observatory for eHealth [11]
- Trial participants were patients suffering from NCDs as defined by the WHO [20]
- The study was published in English or German
- The study was published before August 2015 (no limit concerning the start date)

### Literature search method

An initial systematic literature search was performed between December 2013 and February 2014 in MEDLINE (PubMed), CENTRAL and Business Source Complete. An update of the search was performed in August 2015.

After piloting appropriate search words, the terms were constructed around (1) “mHealth”, (2) “Low and Middle Income Countries” and (3) “Non Communicable Disease”. Search terms for the operationalization of NCDs were derived from WHO’s Global Burden of Disease Report. In addition to the medical terms specified in the Global Burden of Disease Report (e.g., myocardial infarction or dermatological cancer), we added more common terms such as heart or skin (for including interventions against skin cancer) to the search algorithm.

The search conducted in CENTRAL is shown in the Additional file 1: Table S1. It was carried out using the free text search with Boolean operators and MeSH descriptors using the terms Telemedicine [MeSH] AND Developing Countries [MeSH] (with no filter for diseases and the enabled option of exploding all trees). The same search-approach was applied using MEDLINE. Due to a low number of results in the database Business Source Complete it was feasible to exclude the field of terms for NCDs and to include solely the location and intervention of interest.

In addition, reference lists of included studies and identified existing reviews were screened for relevant titles.

After removal of duplicates, the resulting list of titles (Medline 730, CENTRAL 116, Business Source Complete 125) was screened and studies whose titles/abstracts clearly indicated that they were not concerned with mHealth intervention trials for NCDs in LaMICs (e.g., if titles indicated that they focused on developed countries or HIV) were excluded from further consideration.

Full-text articles of 114 studies were retrieved and assessed, resulting in 8 articles included for this review. The screening process was conducted independently by two reviewers (VS and DO). Disagreements were discussed between authors and resolved by consensus.

### Data collection and analysis

For each included study, information was collected on key characteristics of the RCTs concerning:

1. the study location (country, urban/rural);
2. the population (disease, inclusion and exclusion criteria for trial participants);
3. the intervention characteristics, including information on the type of mHealth intervention (e.g., text message, phone call), the data transmitted (e.g., appointment reminders, advice and medication reminders), interactivity of the intervention (i.e., whether it was possible for patients or providers to respond to information received), and personalization (i.e., whether timing or content of information were specific for the patient);
4. the comparator (control) group intervention (e.g., booklet with information on asthma instead of text message with information); and
5. outcomes reported by the studies, including clinical outcomes, compliance, quality of life, costs and other outcomes.

In order to assess the relationship between intervention characteristics and outcomes, studies were categorized into one of four types of mHealth interventions as suggested by Howitt et al. [21] (with slight modifications). We distinguished between interventions for 1) health promotion & awareness, 2) remote monitoring & care support, 3) disease surveillance & outbreak detection, and 4) decision support system.

Meta-analytic techniques were not employed because differences between studies concerning the type of intervention, the included study participants (different diseases), and the reported outcome measures (clinical outcomes, compliance, etc.) made a meaningful analysis of pooled data impossible.

### Risk of bias

Risk of bias was assessed qualitatively concerning selection bias (sequence generation and allocation sequence concealment), performance bias (blinding of participants and personnel), detection bias (blinding of outcome assessment), extent of loss to follow-up, reporting bias (selective outcome reporting), and other bias (e.g., imbalance in baseline characteristics). We used the Cochrane Collaboration’s tool for assessing risk of bias and information on assessment were derived from the text [22]. The full risk assessment of the included studies is available in the Additional file 2.

## Results

### Literature search results

**Fig. 1:**
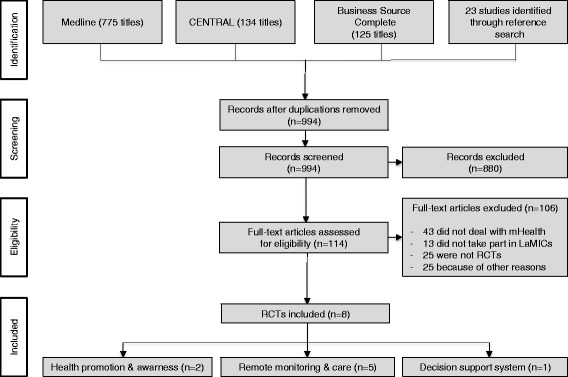
Overview of the screening process

Figure 1 illustrates the literature search and selection process, and presents reasons for exclusion of studies. We identified a total of 969 studies in the three databases and 23 studies were retrieved from references of other studies. Full texts of 114 studies were screened of which 106 were excluded, mostly because they did not deal with mHealth (*n* = 43), did not report results of an RCT (*n* = 25), did not take part in LaMICs (*n* = 13) or because of other reasons (*n* = 25). The final analysis included 8 studies, which met all inclusion criteria.

### Characteristics of included studies

#### Trial characteristics

Table 1 summarizes the main characteristics of the eight included trials. Five studies were conducted in lower middle income countries (LMICs), three in upper middle income countries (UMICs). Two studies [23, 24] reported results of trials, which included patients in both a LMIC and a UMIC (Mexico and Honduras, and India and China, respectively). The participating patients came mostly from urban areas and were recruited mainly from primary care centers or urban hospitals. Three studies dealt with diabetes [25–27], two with asthma patients [28, 29], two with patients suffering from cardiovascular diseases, [23, 24] and one with patients having different NCDs [30], including hypertension, asthma and diabetes.

**Table 1:** Characteristics of the interventions

	Intervention					
Study name	Used channel	Received information	Control group	Timing	Interactivity	Personalization
Balsa and Gandelman [25]	Internet platform & text messages	New topics about type 2 Diabetes and healthy lifestyle	Brief educational brochure	Not reported	No	No
Shetty et al. [26]	Text messages	Medical nutrition therapy, physical activity and drug intake reminders	Oral advises on diet modification and physical activity	Once in three days	No	No
Liew et al. [30]	Text messages	Appointment reminder	No reminder	Once; 24–48 h before the scheduled appointment	No	Yes
Liu et al. [28]	Interactive software on cellphone	Adjustments of therapy	Booklet for written asthma diary and action plan	Immediately after the data has been uploaded	Yes	Yes
Ostojic et al. [29]	Text messages	Adjustments of therapy	No weekly therapeutic advise	Weekly	Yes	Yes
Piette et al. [23]	Mobile blood pressure monitor & phone calls	Advises and medication reminder	No weekly therapeutic advise	Weekly	Yes	Yes
Shahid et al. [27]	Glucometer & Phone calls	Adjustments of therapy	Self monitoring with Glucometer and regular follow up after 4 months	Every 15 days	Yes	Yes
Tian et al. [24]	Smartphone application	Advises on medication prescription and lifestyle changes	Usual cardiovascular management programs	Monthly	No	Yes

A total of 4375 participants were included in all eight studies, of whom 2095 received a mHealth intervention, 314 received an alternative landline-telephone based intervention, and 1966 were included in the control group. Trial size varied from 16 participants [29] to 2086 participants [24]. The mean age in the intervention group was 57.2 years and in the control group 57.8 years. Studies reported a wide range of outcomes, which were classified for the purposes of our review into disease specific clinical outcomes, compliance and others.

#### Intervention characteristics

Table 2 provides an overview about the main characteristics of the mHealth interventions that were evaluated in the eight RCTs. Two interventions informed patients with diabetes about the management of the disease and gave general advice on a healthy lifestyle (category health promotion & awareness): One informed the participants through an internet webpage and frequently sent text messages [25], while the other sent a text message once in three days to the study participants [26]. Both interventions were not personalized to the participants and not interactive.

**Table 2:** Study design characteristics of included RCTs

Study	Location	Income group	Conditions	Place of recruitment	Inclusion criteria	Sample size	Mean Age (Intervention; control)	Planned Follow-up	Measured outcomes
Balsa and Gandel-man [25]	Uruguay (urban)	UMIC	Type 2 Diabetes	Waiting rooms of internists treating diabetic patients at three HMOs in Montevideo	Adult patients with Diabetes 2; Access to Internet (at least once a week)	195 (intervention) 193 (control)	n/d	6 months	Clinical, Others
Shetty et al. [26]	India (urban)	LMIC	Diabetes	Patients at a diabetes centre in Chennai	Type 2 Diabetes with a minimum duration of 5 years; Minimum of high school Education; HbA1c value ranging between 7 % to 10 %	110 (intervention) 105 (control)	50.1; 50.5	1 year	Clinical, Compliance
Liew et al. [30]	Malaysia (urban)	UMIC	Different chronic diseases (mainly NCDs)	Two primary care clinics in Kuala Lumpur	Registered with the clinics for at least 6 months; return appointment between 1 and 6 months; ownership of a mobile phone	314 (telephone) 398 (text mesages) 309 (control)	57.7; 58.1; 60.7	At least 6 months	Compliance
Liu et al. [28]	Taiwan (urban)	UMIC	Asthma	Outpatient clinics of Chang Gung Memorial Hospital, Linkou, northern Taiwan	Moderate to severe Asthma	43 (intervention) 46 (control)	54; 50	6 months	Clinical, Compliance, QoL
Ostojic et al. [29]	Croatia (urban)	UMIC	Asthma	General Hospital “SvetiDuh”, Zagreb	Moderate Asthma for at least 6 months; consistent access to a cellphone, able to use text messages	8 (intervention) 8 (control)	24.5; 24.8	16 weeks	Clinical, Compliance, Costs
Piette et al. [23]	Honduras (rural), Mexico (urban)	UMIC, LMIC	Hypertension	Four private and two public clinics in Cortes, Honduras and one primary care center in Real de Monte	SBP > = 130 mm Hg if diabetic and SBP > = 140 mm Hg if non-diabetic; between 18 and 80 years; access to a cellphone and able to use it	89 (intervention) 92 (control)	58.0; 57.1	6 weeks	Clinical, Others
Shahid et al. [27]	Pakistan (rural)	LMIC	Diabetes	Department of Endocrinology, Liaquat National Hospital	Patients between 18–70 years, residing in rural areas of Pakistan, HbA1c ≥ 8.0 % and having personal functional mobile phone	220 (intervention) 220 (control)	48.95; 49.21	6 months	Clinical, Compliance
Tian et al. [24]	China (rural), India (rural)	UMIC, LMIC	Cardiovascular Diseases	CHWs at 27 villages from 15 townships in China and 20 villages in Haryana State, India	High cardiovascular risk individuals: above 40 years and a self-reported history of coronary disease	1095 (intervention); 991 (control)	59.7; 60.4	One year	Clinical, Compliance

The most basic intervention in the category of remote monitoring & care support was an appointment-reminder system, where text messages were sent 24–48 h before the patients’ scheduled appointments [30].

Four interventions required the patients to record key parameters of their disease, e.g., the Peak Expiratory Flow Rate (PEFR) for patients with Asthma [28, 29], the blood glucose level for patients with Diabetes [27] or the blood pressure for patients with hypertension [23]. They did so by using additional devices (home blood pressure monitor, glucometer, peak expiratory flow meter) and the patients were then asked to send this data either via a text message to a physician [29], to type their records into an interactive phone software [28] or they were called by a specialist and transmitted the information via a phone-call [23, 27]. In all the four studies, patients received personalized disease-management advice.

Only one intervention fell into the category of clinical decision support systems [24]. Community health workers (CHWs) treating patients with cardiovascular diseases in rural areas received a smartphone with an application consisting of prompts regarding the patients’ clinical values, adherence to treatment and other parameters. The application was tailored to the local customs.

### Results of the RCTs

Table 3 provides an overview of all relevant outcomes reported by the eight included studies, illustrating significance of differences in outcomes between the intervention and control groups. The eight studies reported results for a total of 15 different measures of clinical outcome 9 measures of compliance, 2 measures of quality of life (QoL) and 13 other outcome measures.

**Table 3:** Overview of intervention-group outcomes compared to control-group outcomes

Study	Balsa and Gandelman [25]	Shetty et al. [26]	Shahid et al. [27]	Ostojic et al. [29]	Liu et al. [28]	Piette et al. [23]	Liew et al. [30]	Tian et al. [24]
Intervention	Health promotion & awareness		Remote monitoring & care support					Decision support system
Personalization	No		Yes					
Interactivity	No		Yes				No	
Disease	Diabetes			Asthma		Hyper-tension	Various NCDs	CVDs
Clinical outcomes								
SBP^a^ (mm Hg), Mean	+/−		++			+ / ++^b^		++
Fasting blood glucose level	+/−							
BMI^c^, kg/m^2^		+/−^d^	+/−^e^					
PPG^f^ < 180 mg		++						
HbA1c^g^		++^h^	++^i^					
TC^j^ < 150 mg/dl		++						
HDL-C^k^ > 40 mg/dl		+/−						
LDL-C^l^ < 100 mg		++	++					
FEV1%^m^, predicted				+	++			
PEFR^n,^ L/min				+	++			
PEFRvariability				++				
Coughing				++				
Night symptoms				++				
Wheezing				+/−				
Limitation of activities				+/−				
Compliance outcomes								
Attendance		+					++	
ICS^o^ dosage				+/−	+			
Systemic steroids				+/−	+			
Antileukotrienes				+/−	+/−			
Long-acting beta2-agonist								
Anti-hypertensive medication use								++
Aspirin								++
Adherence to diet prescription		+/−	++					
Adherence to physical activity		+	++					
Quality of life related outcomes								
Physical component					++			
Mental component					++			
Cost								
Monetary				-				
Timely				-				
Other outcomes								
Knowledge	+/−							
Perception of health quality	+/−							
Health-related behaviors	+/−							
Physician-Patient relationship	+/−							
Number of visits to emergency department					++			
Depression scores						++		
Perceived overall health						++		
Overall satisfaction with care						++		
Medication problems						++		
Current smoker, %								+/−
Awareness of harms of high salt diet, %								+/−
Receiving monthly follow-up, %								++
Hospitalization during the past year, %								+

(+/−): no difference; (+): superior to control group without significance; (++): superior to control group with significance (*p* < 0.05); (−): inferior to control group. A more detailed summary of reported outcomes, specifying values for intervention and control groups is available in Stephani et al. [44]

^a^Systolic Blood Pressure

^b^Subgroup of low-literacy people/people with higher education needs

^c^Body Mass Index

^d^BMI < 26

^e^BMI < 25

^f^Postprandial Plasma Glucose Test

^g^Glycated hemoglobin

^h^HbA1c < 8 %

^i^mean HbA1c level

^j^Total Cholestorol

^k^High-Density Lipoprotein Cholesterol

^l^Low Density Lipoprotein

^m^Peak Expiratory Flow Rate

^n^Forced Expiratory Volume in 1 second

^o^Inhaled Corticosteroid

The two *health promotion & awareness* interventions targeted diabetic patients but none of the reported outcome measures was available from both studies. In the study by Balsa and Gandelman [25], where diabetic patients received a text message that intended to motivate their use of a website, neither clinical outcomes nor other outcomes were improved. In the study by Shetty et al. [26], where patients received a text message with advice on nutrition, physical activity and drug intake, several clinical outcome measures showed significant improvements, although compliance measures did not improve significantly.

Out of the five studies evaluating tools for *remote monitoring and care support*, one study evaluated an interactive telephone-intervention for patients with diabetes [27]. Patients were advised to self-monitor their Blood Glucose level and received therapeutic advice over the phone twice a month. The study found that clinical and compliance outcomes improved significantly more strongly in the intervention group than in the control group.

Two studies evaluated interactive mHealth interventions for patients with asthma [28, 29], where patients transmitted information about their pulmonary function (as assessed by the peak expiratory flow rate, PEFR) to a physician and received personalized therapeutic advice (e.g., medication adjustments). Both studies found that individuals showed improved pulmonary function tests (FEV1% predicted and PEFR), although this finding was not significant in the study by Ostojic et al. [29], which included only a total of 16 participants. Liu et al. [28] also found significant improvements of quality of life, while Ostojic et al. [29] found significant improvements of PEFR variability and of some self-reported clinical outcome measures (e.g., coughing and night symptoms) although not of others, and no effect on compliance.

Piette et al. [23] found that their intervention providing personalized advice to hypertensive patients on the basis of their self-recorded blood pressure lowered systolic blood pressure in the intervention group, although this finding was significant only in a subgroup of 117 out of 181 participants with low literacy or high hypertension information needs.

Liew et al. [30] found that text messages and telephone appointment reminders lowered non-attendance of patients significantly when compared to controls.

The only study of a decision support system by Tian et al. [24] found that medication compliance of patients treated by CHWs, who were supported by smartphones, increased significantly, and they had significantly lower blood pressure when compared with controls.

The impact of mHealth on **costs** in terms of time and money for physicians and patients was observed by only one trial [29]. It was estimated that the intervention would lead to additional monetary costs per patient of €0.67 per week for text messages sent to physicians, and that physicians spent 2 min per patient per week at a cost of 1 Euro per patient.

## Discussion

This is the first review focusing specifically on RCTs of mHealth interventions against NCDs in LaMICs. Despite much enthusiasm about the ‘great potential’ of mHealth for addressing NCD needs in LaMICs and despite a growing body of literature on the topic, we found only eight studies that reported results of RCTs performed in LaMICs. Except for one study [25], these showed generally positive effects of mHealth interventions on reported outcome measures. However, because trials tested different interventions (ranging from health promotion over appointment reminders and medication adjustments to clinical decision support systems), and included patients with different diseases (diabetes, asthma, hypertension), and – partially as a result of this – reported very different outcome measures, it is impossible to generalize these findings.

Nevertheless, our review provides a first glimpse of the slowly emerging evidence base on the effectiveness of mHealth interventions for NCDs and has important implications for policy-makers and researchers. First, it is remarkable that the evaluated mHealth interventions generally showed positive effects on reported outcome measures, including clinical outcomes, compliance, and quality of life. This finding is in line with findings from a much broader literature on communicable disease and maternal care, where many different kinds of mHealth interventions have been found to improve clinical outcomes and compliance of patients – although results have been shown to vary depending on the specific type of intervention [31–33].

Second, our results suggest that particular types of mHealth interventions that were found to have positive effects on patients with communicable diseases and for improving maternal care are likely to be effective also for NCDs. For example, text message appointment reminders have been found to lead to higher pre-natal visit rates of pregnant women [34–36], and two studies included in our review show that they are also effective at increasing attendance rates of patients with NCDs [26, 30]. Similarly, drug intake reminders have been found to improve treatment adherence of people with AIDS and Malaria [37–39], and one study in our review showed that drug intake reminders (combined with other information on medical nutrition and physical activity) improve clinical outcomes of patients with Diabetes [26].

Third, our results show that there is very limited evidence on the effects of mhealth in low income countries as all included studies reported results of trials conducted in middle income countries. Furthermore, when considering the 4 broad categories of mHealth interventions that we defined at the beginning, i.e., interventions of 1) health promotion & awareness, 2) remote monitoring & care support, 3) disease surveillance & outbreak detection, and 4) decision support system, it is evident that available RCTs have focused mostly on mHealth interventions falling into category 2. Also Bloomfield et al. [16] concluded that there is very limited evidence concerning a wide range of health systems challenges, which could potentially be addressed by the implementation of mHealth interventions. In our review, several studies evaluating *clinical decision support* systems were identified during full-text screening [40–43] but they had to be excluded because they were no RCTs. Information on cost-effectiveness of mHealth interventions is largely unavailable and only one study included in our review considered the effect of mHealth on costs of care [29].

An important limitation of our review is that we excluded all studies that did not report results of RCTs. Observational studies and non-randomized trials may provide important bits of information that are useful for understanding the effectiveness of mHealth. Nevertheless, we opted for excluding these studies as non-randomized trial designs carry a greater risk of being flawed as a result of multiple biases [22]. Another limitation of the review process could have been the restriction to the two languages German and English. Furthermore, given the limited number of studies, it was not possible to compare results of different studies. Effects of mHealth are likely to differ depending on the specific type of intervention, the specific disease, and the specific context. Consequently, it is impossible to draw firm conclusions on the effectiveness of mHealth interventions in general, e.g., by carrying out pooled analyses of outcome data. Finally, the specific effects of different kinds of mHealth interventions on different kinds of patients with NCDs living in LaMIC could not be investigated. For example, it is likely that the effectiveness of interventions depends on whether patients can interact with health professionals and whether information is personalized to the patients. Although our review includes studies with both interactive and non-interactive interventions as well as studies with both personalized and non-personalized information, the specific effects of these different interventions could not be compared because they were provided to different patients (in difference settings) and reported different outcome measures.

## Conclusion

Our review shows that there are only eight studies reporting results of RCTs of mHealth interventions for patients with NCDs in LaMICs. These have generally found positive results. However, a more detailed analysis of the specific effects of different types of mHealth interventions on different types of patients and a firm conclusion about the effectiveness of mHealth against NCDs is impossible because of the small number of studies and the heterogeneity of reported outcome measures.

Nevertheless, our results indicate that some findings of the positive effects of mHealth interventions for patients with communicable diseases and for maternal care can be replicated by mhealth interventions for patients with NCDs. However, we can only repeat the conclusions of previous reviews [15, 16] that more research is needed to fill the many gaps in knowledge about mHealth interventions for NCDs in LaMICs.

## Abbreviations

CHWs, community health workers; DALYs, Disability Adjusted Life Years; FEV1%, Forced Expiratory Volume in 1 second; LaMICs, low and middle income countries; LMICs, lower middle income countries; mHealth, mobile health; NCDs, non-communicable diseases; PEFR, peak expiratory flow rate; RCTs, randomized controlled trials; UMICs, upper middle income countries

## Additional files

Additional file 1: Table S1. Search method conducted with the CENTRAL-database (DOC 30 kb) Additional file 2: Table S2. Bias of the included studies (DOC 34 kb)
