# Supplementary material for: A systematic review of randomized controlled trials of mHealth interventions against non-communicable diseases in developing countries
Source: BMC Public Health. 2016 Jul 15;16:572. doi: 10.1186/s12889-016-3226-3 (PMC4946127; doi:10.1186/s12889-016-3226-3)
Supplement: Additional file 1: Table S1. — Search method conducted with the CENTRAL-database (DOC 30 kb) [file 12889_2016_3226_MOESM1_ESM.doc]

**Additional table : Search method conducted with the CENTRAL-database**

| **ID** | **Search** |
| --- | --- |
| #1 | MeSH descriptor: [Telemedicine] explode all trees |
| #2 | MeSH descriptor: [Developing Countries] explode all trees |
| #3 | #1 and #2 |
| #4 | (”m-Health” or ”mHealth” or mobile or SMS or tele*) |
| #5 | ”developing countries” or ”developing world” or ”rural areas” or  ”low-resource” or ”low-income” or ”mid-income” |
| #6 | ”non-communicable” or noncommunicable or chronic or cancer or neoplasms or diabetes or mental or neurological or ”sense organ” or ophthalmology or cardiology or cardiovascular or heart or digestive or genitourinary or skin or dermatology or musculoskeletal or congenital or oral or pathology or psychiatry or “chronic respiratory disease” |
| #7 | #4 and #5 and #6 |
| #8 | #7 or #3 |
