# Supplementary material for: A systematic review of randomized controlled trials of mHealth interventions against non-communicable diseases in developing countries
Source: BMC Public Health. 2016 Jul 15;16:572. doi: 10.1186/s12889-016-3226-3 (PMC4946127; doi:10.1186/s12889-016-3226-3)
Supplement: Additional file 2: Table S2. — Bias of the included studies (DOC 34 kb) [file 12889_2016_3226_MOESM2_ESM.doc]

| Additional table 2: Bias of the included studies | **Sequence generation – Selection bias** | **Allocation sequence concealment – Selection bias** | **Blinding of participants and personnel – Performance bias** | **Blinding of outcome assessment – Detection bias** | **Incomplete outcome data** | **Selective outcome reporting – Reporting bias** | **Other bias** |
| --- | --- | --- | --- | --- | --- | --- | --- |
|  |  |  |  |  |  |  |  |
| **Balsa and Gandelman [24]** | +/- | +/- | +/- | +/- | + | + | + |
| **Liew et al. [29]** | + | + | + | + | + | + | + |
| **Liu et al. [27]** | +/- | +/- | +/- | +/- | - | + | + |
| **Osotjic et al. [28]** | + | +/- | + | + | + | + | + |
| **Piette et al. [22]** | + | +/- | - | +/- | + | + | +/- |
| **Shahid et al. [26]** | - | +/- | +/- | + | + | + | + |
| **Shetty et al. [25]** | + | +/- | +/- | +/- | - | +/- | + |
| **Tian et al. [23]** | + | +/- | +/- | + | + | + | - |

(+/-): unclear risk of bias; (-): high risk of bias; (+): low risk of bias
